# HBx inhibits DNA sensing signaling pathway via ubiquitination and autophagy of cGAS

**DOI:** 10.1186/s12985-022-01785-3

**Published:** 2022-03-28

**Authors:** Hong Chen, Linshan Jiang, Shu Chen, Qin Hu, Ying Huang, Ying Wu, Weixian Chen

**Affiliations:** 1 Department of Laboratory Medicine, The Second Affiliated Hospital of Chongqing Medical University, No.74 Linjing Road, Yuzhong District, Chongqing, 400010 China; 2 Department of Infectious Diseases, The Second Affiliated Hospital of Chongqing Medical University, Chongqing, China; 3 Clinical Medicine Research Centre, Liuzhou People’s Hospital, Guangxi Medical University, Liuzhou, China

**Keywords:** cGAS, HBx, IFN-β, Ubiquitination, Autophagy

## Abstract

**Background:**

Cyclic GMP-AMP synthase (cGAS) is a crucial DNA sensor and plays an important role in host antiviral innate immune responses. During hepatitis B virus (HBV) infection, the cGAS signaling pathway can suppress HBV replication. As an important regulatory protein of HBV, hepatitis B virus X protein (HBx) may serve as an antagonistic character to the cGAS/STING signaling pathway. In this study, we aim to investigate the functional role of HBx in the cGAS/STING signaling pathway.

**Methods:**

The effects of HBx on IFN-β promoter activity were measured by Dual-luciferase reporter assays. Ubiquitination and autophagy were analyzed by Western-blot and Co-immunoprecipitation assays.

**Results:**

Our results show that HBx down-regulates IFN-I production by directly promoting ubiquitination and autophagy degradation of cGAS.

**Conclusions:**

HBV can antagonize host cGAS DNA sensing to promote HBV replication and provide novel insights to develop novel approaches against HBV infection.

## Introduction

The innate immune system constitutes the first line of host defense against invading pathogens and recognizes pathogen-associated molecular patterns through interacting with various pathogen recognition receptors (PRRs), then triggering the production of type I interferon (IFN-I), tumor necrosis factor, and other antiviral factors [1, 2]. cGAS serves as a cytosolic DNA sensor belonging to the PRR, which catalyzes the synthesis of cGAMP and induces the activation of transcription factor interferon regulatory factor-3 (IRF-3), subsequently promoting IFN-I release and IFN stimulated genes (ISGs) expression such as ISG15, ISG56 [3, 4]. Besides, cGAS exhibits antiviral activity against a series of DNA and RNA viruses, such as herpes simplex virus 1 (HSV-1), dengue virus (DENV), and HIV [5–7]. Therefore, it is widely accepted that cGAS is involved in recognizing HBV. Importantly, growing evidence indicates that the cGAS-mediated signaling pathway promotes innate immune response against HBV.

Furthermore, several research groups reported that HBV replication could be inhibited by cGAS. In brief, cGAS is shown to recognize HBV DNA and is required to initiate the antiviral innate immunity to suppress HBV replication [8–10]. Since activation of cGAS elicits a potent antiviral response, HBV may enable its maintenance in infected hepatocytes by employing strategies to attenuate the cGAS-STING signaling cascade.

HSV-1 tegument protein VP24, UL41, UL24, UL46, UL36, US3, and VP22 were demonstrated to evade the cGAS/STING-mediated signaling pathway[11–17]. On the other hand, Kaposi's sarcoma-associated herpesvirus (KSHV) ORF52 suppresses cytosolic DNA sensing by directly inhibiting cGAS enzymatic activity through cGAS binding and DNA binding [18]. KSHV's N-terminal domain of the latency-associated nuclear antigen (LANA) interacted directly with cGAS, thereby antagonizing cGAS and inhibiting the subsequent 2′3' cGAMP and IFN-I production[19]. However, the underlying mechanisms of HBV evading innate immune responses are still poorly understood. cGAS could sense the naked relaxed-circular HBV DNA but fail to sense the nucleic acids during HBV infection as the genome is likely packaged to the viral capsid. Nevertheless, it is reported that HBV infection suppresses the expression and function of cGAS [20]. However, studies are needed to elucidate the potential mechanisms responsible for suppressing cGAS by HBV.

HBV is a hepatotropic, non-cytopathic, enveloped, and partially double-stranded DNA (dsDNA) (3.2 kb) virus classified into the Hepadnaviridae family[21], which can be sensed by the cGAS/STING signaling pathway and activate the host antiviral responses. HBx is the unique non-structure protein of HBV and encoded by one of the four open reading frames of HBV. It is well known as an important coactivator for HBV replication and HBV-associated hepatocellular carcinogenesis [22]. HBV is also regarded as a stealth virus because it could invade and replicate efficiently in the human liver by escaping from the host's innate antiviral immunity. Recently, a novel viral strategy has been reported that HBV suppresses the expression and function of cGAS to evasion from the innate immune responses activated by the cGAS/STING signaling pathway[20]. However, the specific mechanisms involving HBV inhibiting the expression and function of cGAS have not been fully illustrated. We report here that HBx, as an HBV-specific component protein, downregulates the accumulation of cGAS expression by promoting its ubiquitination and autophagy, thereby escaping the innate immune response activated by the cGAS/STING signaling pathway and establishing an effective infection.

## Methods

### Cell culture and transfection

The human hepatocellular carcinoma cell lines SMMC-7721, LO2, HepG2, HepG2.2.15, and HEK293T, were maintained in the Dulbecco's modified Eagle's medium (DMEM) with 10% FBS (Gibco, USA), 100 U/ml penicillin, and 100 μg/ml streptomycin. The cell culture was maintained at 37 °C in a humid atmosphere containing 5% CO2. Transient transfections were performed with Lipofectamine 2000 according to the manufacturer's protocol.

### Reagents and antibodies

The primary antibodies used for this study were as follows: mouse anti-HBx monoclonal antibody (Santa Cruz, USA), rabbit anti-cGAS monoclonal antibody (CST, USA), rabbit anti-STING monoclonal antibody (CST, USA), rabbit anti-IRF3(Phospho-Ser382) monoclonal antibody (CST, USA), mouse anti-IRF3 monoclonal antibody (CST, USA), mouse anti-β-actin monoclonal antibody (Santa Cruz, USA), mouse anti-GAPDH Monoclonal Antibody(Proteintech, USA), rabbit anti-Flag polyclonal antibody (Proteintech, USA), rabbit anti-HA polyclonal antibody (Proteintech, USA), K48-linked ubiquitin antibody(Billerica, MA, USA), DAPI (Santa Cruz, USA) and Lipofectamine 2000 (Invitrogen, USA) were used following the manufacturer's instructions.

### Luciferase reporter assays

HEK293T cells seeded on 24-well plates were transiently transfected with 200 ng of the luciferase reporter plasmid together with a total of 600 ng of various expression plasmids and/or empty vector controls using Lipofectamine 2000. As an internal control, 50 ng of pRL-TK (Renilla luciferase) was transfected simultaneously. According to the Promega Dual-Luciferase Reporter Assay protocol, luciferase assays were then performed at 24 h post-transfection. The relative luciferase activity was expressed as arbitrary units by normalizing firefly to Renilla luciferase activity.

### Real-time quantitative PCR analysis

Cells were transfected for 24 h and then lysed with Trizol (Invitrogen, Carlsbad, CA, USA). Complementary single-stranded DNA was synthesized from total RNA by reverse transcription (TaKaRa, Japan). Quantification of cDNA targets was performed on CFX96TM Real-Time-PCR Detection System (Bio-Rad, USA). Primers were synthesized by Invitrogen and are listed in Table 1.

**Table 1:** Primers for qRT-PCR

Primer	Sequence
cGAS Forward	5'AAGCTCCGGGCGGTTTTGGA 3'
cGAS Reverse	5'AGGTGCAGAAATCTTCACGTGCTC3'
STING Forward	5'GTGGCTTGAGGGGAACCCGC3'
STING Reverse	5'GGCTGGAGTGGGGCATCTTCT3'
IFN-β Forward	5'AGGACAGGATGAACTTTGAC3'
IFN-β Reverse	5'TGATAGACATTAGCCAGGAG3'
ISG56 Forward	5'TCTCAGAGGAGCCTGGCTAA3'
ISG56 Reverse	5'TGACATCTCAATTGCTCCAG 3'
ISG54 Forward	5'GGAGCAGATTCTGAGGCTTTGC3'
ISG54 Reverse	5'GGATGAGGCTTCCAGACTCCAA3'

### Western blotting

The levels of cGAS, STING, HBx, and IRF3 in cells were evaluated by western blotting. Briefly, samples containing an equal amount of protein were separated by SDS-PAGE and blotted onto PVDF membranes. The membranes were blocked with 5% bovine serum albumin and incubated with anti-cGAS, anti-STING, anti-HBx, anti-IRF3, anti-Flag, anti-HA, anti-K48-linked ubiquitin, or anti-β-actin antibodies, followed by incubation with secondary antibodies conjugated with horseradish peroxidase. The proteins of interest were detected using the SuperSignal West Pico Chemiluminescent Substrate kit (Thermo Fisher Scientific, USA). The results were recorded by the Bio-Rad Electrophoresis Documentation (Gel Doc 1000, Bio-Rad, USA) and Quantity One Version 4.5.0.

### Native PAGE

Native polyacrylamide gel electrophoresis (PAGE) was performed using ReadyGels (7.5%, Bio-Rad). In brief, gels were prerun with 25 mM Tris and 192 mM glycine, pH 8.4, with 1% deoxycholate (DOC) in the cathode chamber for 30 min at 40 mA. Samples in native sample buffer (10 g of protein, 62.5 mM Tris–Cl, pH 6.8, 15% glycerol, and 1% DOC) were size-fractionated by electrophoresis at 25 mA, and the gel was transferred to nitrocellulose membranes for WB analysis.

### Statistical analysis

The Student's t-test was used for all statistical analyses with GraphPad Prism 5.0 software(GraphPad Software, CA, USA). Differences between groups were considered significant when the P-value was less than 0.05. Statistical differences were shown to be at different levels of *P* < 0.05 (∗) and *P* < 0.001 (∗∗∗).

## Results

### Hepatitis B virus X protein is a negative regulator of the cGAS-induced IFN-I signaling pathway

**Fig. 1:**
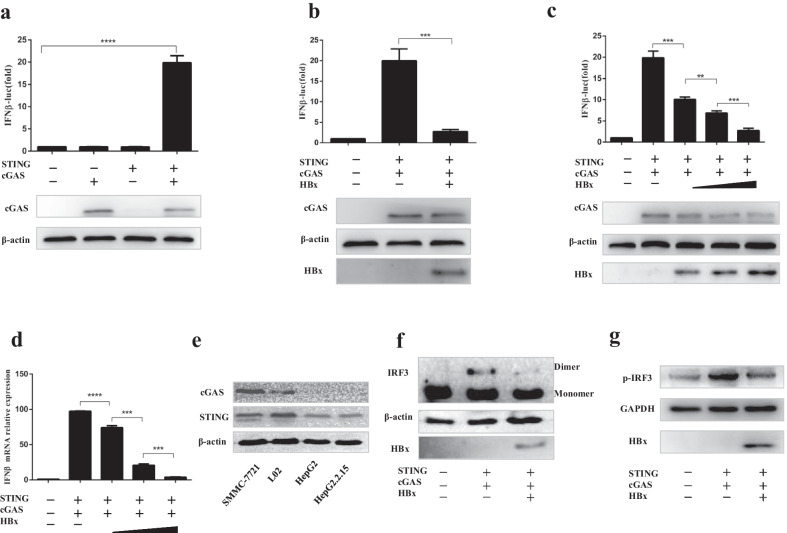
Hepatitis B virus X protein is a negative regulator of cGAS-Induced IFN-I-β signaling. (**a**–**c**) HEK293T cells were transfected with IFN-I-β luciferase reporter and expression plasmids as indicated, and luciferase activity was assayed 24 h after transfection. The relative luciferase activity was expressed as arbitrary units by normalizing firefly luciferase activity to Renilla luciferase activity. (**d**) HEK293T cells were transfected with expression plasmids as indicated, at 24 h posttransfection, cells were harvested and subjected to qRT-PCR analysis. (**e**) Western blot (WB) analysis of cGAS, STING in several human hepatic cell lines. (**f**, **g**) HEK293T cells were cotransfected with cGAS and STING, along with empty vector or HBx-Flag plasmid, Western blot analysis of IRF3 dimerization, and phosphorylation

Currently, substantial evidence demonstrates that cGAS recognizes cytosolic DNA, leading to IFN-I production. HBx is an enigmatic molecule because of its pleiotropic functions in regulating IFN-I production[23]. To evaluate the effect of HBx on the cGAS/STING signaling pathway during DNA virus infection, we carried out dual-luciferase reporter (DLR) assays to detect interferon beta (IFN-β) promoter activity. In HEK293T cells, transfection with cGAS or a minimal amount of STING alone could not activate the IFN-β promoter, whereas cotransfection of cGAS and STING plasmids significantly activated the IFN-β promoter (Fig. 1a). Then, HEK293T cells were cotransfected with cGAS, STING, and IFN-β luciferase reporter in the presence or absence of HBx. The results showed that HBx could significantly inhibit the IFN-β promoter activity in a dose-dependent manner (Fig. 1b, c). We further measured the mRNA of IFN-β to validate the expression level, and similar results were obtained (Fig. 1d).

Next, cGAS and STING expression levels were detected in several hepatic cell lines, including HepG2, HepG2.2.15, L02, and SMMC-7721. The results indicated that STING was highly expressed in all the cell lines. However, we only detected the expression of cGAS in L02 and SMMC-7721 cells (Fig. 1e).

It is reported that cGAS senses cytosolic DNA and catalyzes the synthesis of cGAMP, which subsequently binds to STING, leading to IRF3 dimerization and phosphorylation, finally producing IFN-β [3, 24]. Previous studies have reported that HBx could suppress virus-triggered IRF3 activation through the act on MAVS to inhibit the expression of IFN-β [25]. Dimerization and phosphorylation are the activated state of IRF3, and we verified that HBx could suppress cGAS-triggered dimerization and phosphorylation of IRF3 to inhibit IFN-β (Fig. 1f, g).

### HBx inhibits the IFN-I signaling pathway upstream of STING

**Fig. 2:**
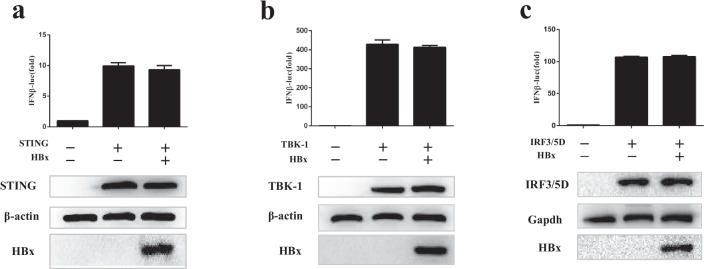
HBx inhibits the IFN-I-β signaling pathway at the level upstream of STING. (**a**–**c**) HEK293T cells were cotransfected with expression plasmids as indicated, and cells were harvested 24 h after transfection and subjected to DLR assay. The expression of STING, IRF3/5D, TBK1, and HBx was analyzed by WB analysis. The data represent results from one of the triplicate experiments. Error bars represent standard deviations of three independent experiments

To clarify what level HBx acts on the cGAS-STING pathway to block the production of IFN-β, we combined HEK293T cells with empty vectors or HBx plasmids, as well as IFN-β-Luc reporter genes and important adaptor proteins in the expression pathway, including the active forms of STING, tank-binding kinase 1 (TBK1) and IRF3 (IRF3/5D). We found that ectopic expression of HBx does not affect the activation of IFN-β promoters driven by STING, TBK1, or IRF3/5D (Fig. 2). These results suggest that HBx can inhibit the expression of IFN-I-β upstream of STING.

### HBx inhibits the cGAS protein level

**Fig. 3:**
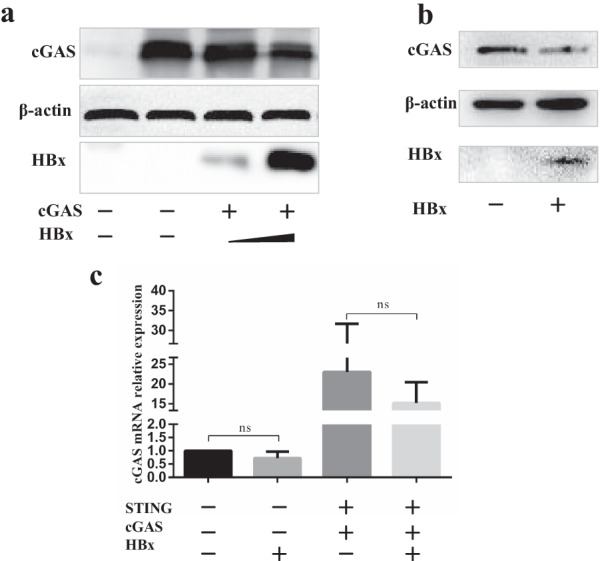
HBx down-regulates the cGAS expression and function. (**a**) HEK293T cells were transfected with cGAS expression plasmids, and then cells were harvested 24 h postinfection and subjected to WB analysis. (**b**) SMMC-7721 cells were transfected with expression plasmids as indicated at 18 h posttransfection, and cells were harvested and subjected to qRT-PCR analysis. The data represent results from one of the triplicate experiments. Error bars represent the SDs of three independent experiments

The above mentioned data led us to hypothesize that HBx might mainly act directly on cGAS. KSHV virion protein ORF52 inhibits cGAS enzymatic activity [18]. HSV-1 tegument protein UL41 downregulates the expression of cGAS, and VP22 interacts with cGAS directly to interfere with its DNA sensing [12, 17]. Previous research has shown that HBV infection suppresses the expression of cGAS and its related genes [20]. HEK293T cell line was used as a cell model to explore whether the role of HBx is consistent with HBV and if HBx is the active component that directly targets the cGAS. Ectopic expression of cGAS in HEK293T was performed to test whether the HBx could decrease the expression level of cGAS. HEK293T cells were ectopically expressed cGAS, harvested, and subjected to Western blot analysis. As shown in Fig. 3a, HBx down-regulated the expression of cGAS in a dose-dependent manner (Fig. 3a). Previous experiments have shown that SMMC-7721 cells contain endogenous cGAS.SMMC-7721 cells were transfected with HBx plasmid, harvested, and subjected to Western blot (WB) analysis. As shown in Fig. 3b, HBx significantly downregulated the expression of endogenous cGAS (Fig. 3b). Interestingly, the cGAS protein level decreased markedly when HBx expression increased. However, the abundance of cGAS mRNA did not change with the increased expression of HBx (Fig. 3c). Notably, we observed that HBx did not affect the transcriptional level of cGAS. Further study is required for the regulation mechanism of HBx acting on cGAS.

### HBx binds to and interacts with cGAS

**Fig. 4:**
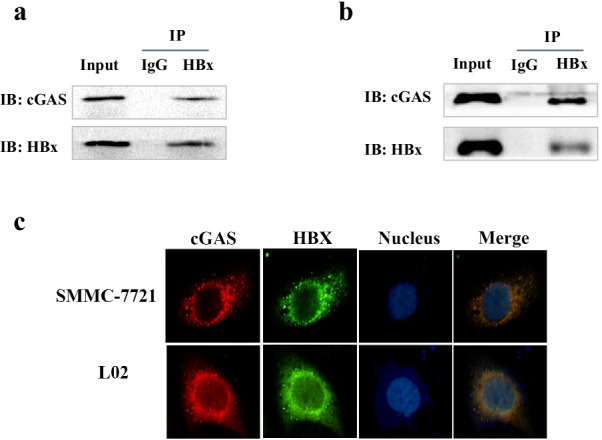
HBx binds to and interacts with cGAS. (**a**, **b**) SMMC-7721 cells were transfected with an empty vector or HBx-Flag plasmid. At 24 h posttransfection, cells were harvested, and obtain total protein. We used the IgG and anti-HBx or anti-cGAS antibody to pull the protein, and the interaction was detected by western blot analysis. (**c**) SMMC-7721 and L02 cells were transfected with an empty vector or HBx-Flag plasmid. At 24 h posttransfection, stain with fluorescent antibody as indicated and observed by fluorescence microscope

It is known that DNA binds to the N terminus of cGAS to promote cGAS activation [24]. In gammaherpesviruses, ORF52 blocks cGAS activity in part through their interaction. The specific inhibition of cGAS by ORF52 prompted us to investigate the possibility of an interaction between cGAS and HBx. Previous reports have revealed that HBx inhibited the IFN-β signaling pathway by decreasing the expression and function of cGAS. To further explore the role of HBx in IFN-β production by downregulating the expression of cGAS, SMMC-7721 and LO2 cells were transfected with HBx plasmids, 24 h after transfection, cells were lysed, and coimmunoprecipitation experiments were performed. Expression of the transfected proteins was analyzed by immunoblotting with anti-Flag and anti-cGAS antibodies. As shown in Fig. 4a, HBx bound and interacted with cGAS but not STING.

Meanwhile, HBx was used to pull down cGAS, and a similar result was observed (Fig. 4b). Then fluorescence microscopy also indicated that HBx co-localized with cGAS in various cell types (Fig. 4c). These results suggest that HBx binds to cGAS, which may block DNA recognition by cGAS.

### HBx promotes ubiquitination and autophagy degradation of cGAS

**Fig. 5:**
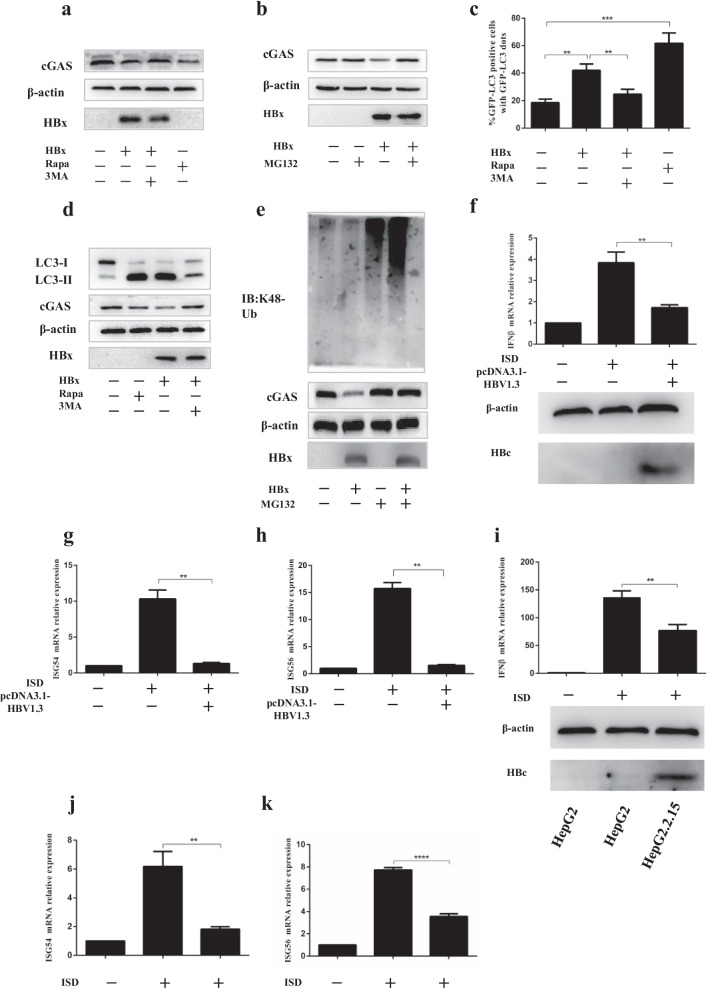
HBx promotes ubiquitination and autophagy degradation of cGAS. (**a**–**b**) SMMC-7721 cells were transfected with an empty vector or HBx-Flag plasmid. At 12 h posttransfection, treated with Rapamycin or 3-MA or MG132 (10 mM) for 12 h, dimethyl sulfoxide (DMSO) was used for controls. Cells were harvested and subjected to WB analysis. (**c**, **d**) SMMC-7721 cells were transfected with pGFP-LC3 and empty vector or HBx- Flag plasmid. At 12 h posttransfection, treated with Rapamycin or 3MA or MG132 (10 mM) for 12 h, dimethyl sulfoxide (DMSO) was used for controls. Moreover, autophagic dots were observed by a fluorescence microscope. Quantitation of the autophagic cells in SMMC-7721. Cells with more than three fluorescent particles are considered autophagy-activated cells. Meanwhile, the cells were harvested and subjected to WB analysis. (**e**) SMMC-7721 cells were transfected with an empty vector or HBx- Flag plasmid. At 12 h posttransfection, cells were harvested and subjected to WB analysis. (**f–h**) L02 were transfected with ISD, empty vector or HBV1.3 plasmid, IFNβ, ISG54, and ISG 56 were detected by qRT-PCR analysis at 12 h posttransfection, HBV core protein was detected by WB analysis. (**i**–**k**) HepG2 cells and HepG2.2.15 cells were transfected with ISD, IFNβ, ISG54, and ISG 56 were detected by qRT-PCR analysis at 12 h posttransfection, HBV core protein was detected by WB analysis

The degradation of protein mainly consists of two pathways: ubiquitin–proteasome and autophagy-lysosome. It is known that HBx has been shown to play a critical role in HBV-mediated autophagy. It is reported that the lysine 48 (K48)-linked ubiquitin chains of cGAS connect with microtubule-associated protein-Light-chain 3 (LC3), which mediate the autophagic degradation of cGAS [26]. Pharmacologic approaches were employed to investigate which pathways participate in regulating the expression of cGAS. We observed that the autophagic enhancer Rapamycin and HBx down-regulated the protein level of cGAS. Besides, both autophagic inhibitor 3-methyladenine (3-MA) and proteasome inhibitor MG132 could up-regulate the protein level of cGAS (Fig. 5a, b).

LC3 from a diffuse cytoplasmic distribution to point aggregation has been used as a marker of autophagy activation [26]. GFP-LC3 fluorescent particles were detected to reflect the autophagy level. We observed that HBx and Rapamycin dramatically increased GFP-LC3 redistribution into aggregation dots, and 3-MA could suppress this phenomenon. The percentage of GFP-LC3-positive cells induced by HBx was increased almost threefold compared to the control (Fig. 5c). Besides, (LC3) transforms from a free form (LC3-I) to a phosphatidylethanolamine-conjugated form (LC3-II) during autophagy. LC3-II also serves as an indicator of autophagy [26]. Our study found that HBx and Rapamycin markedly up-regulated LC3-II expression and reversed the ratios of LC3-I/LC3-II compared to the control (Fig. 5d). Previous studies have shown that cGAS was mainly ubiquitinated with K48 linkage. We also detected the K48-linked ubiquitination of cGAS and found that HBx could promote the K48-linked ubiquitination of cGAS (Fig. 5e). To further verify that HBx is the main component protein that regulates the cGAS/STING pathway of HBV infection, ISD is used to sensitize the cGAS/STING signal channel. HBV 1.3-fold genome plasmid (pcDNA-HBV1.3) was transfected into the L02 cell line to establish an HBV-infected cell model (Fig. 5f–h), HepG2.2.15 is an HBV stably transfected cell line constitutively producing HBV. The mRNA levels of IFN-β, ISG54, and ISG56 were examined in HepG2 and HepG2.2.15 cell lines (Fig. 5i–k). We found that the mRNA levels of IFN-β, ISG54, and ISG56 in HBV positive cells all decreased significantly, compared with the effect of HBx, suggesting that HBx is responsible for HBV to inhibit cGAS/STING signaling pathway. These results showed that HBx as the main component protein of HBV could down-regulate the protein level of cGAS by promoting autophagy and ubiquitination, further inhibiting cGAS mediated pathways, thereby inhibiting the expression of IFN-I-β and IFN stimulated genes, such as ISG56, ISG54.

## Discussion

Two main hepatitis viruses currently cause chronic viral hepatitis, including HCV and HBV. HCV is a single-stranded RNA virus, and the treatment of HCV infection has been overcome. However, HBV is so sneaky that the cure for HBV infection is still limited [27]. HBV is a hepatotropic DNA virus [21]. It is widely accepted that DNA sensors bridge the host sensing DNA virus and induced immune defense during the past decade. HBV infection can be sensed by the DNA sensor of cGAS, which activates the cGAS/STING signaling pathway and induces the innate immune response [9]. HBV is so sly that it prompts us to presume HBV might evolve certain mechanisms to block this signal pathway. cGAS inhibits HBV is widely reported, but the immune evasion of HBV is poorly understood.

There are three main ways to regulate cGAS: (i) post-translational modifications, including phosphorylation, ubiquitination, sumoylation, and glutamylation; (ii) crosstalk with other pathways such as autophagy, inflammasome; (iii) regulation of cGAS by viral proteins [28]. There is growing evidence that various viruses regulate cGAS through these pathways, thereby regulating innate immune response induced by the cGAS signal axis. Kaposi's sarcoma-associated herpesvirus ORF52, HSV-1 tegument protein UL41, VP22, and the N-terminal domain of the latency-associated nuclear antigen were reported to target cGAS directly. Previous studies have shown that cGAS inhibits HBV by multiple strategies. Meanwhile, HBV evades cGAS sensing by decreasing cGAS and its effector gene expression. However, little is known about whether HBV evades the cGAS-STING signaling pathway. For the first time, we reported that HBx is an effective HBV protein targeting cGAS, as HBx could down-regulate cGAS expression and further inhibit IFN-β and ISG56 expression induced by cGAS. However, HBx did not down-regulate cGAS mRNA, indicating that it did not mediate the transcriptional level of cGAS.

Ubiquitin–proteasome and autophagy-lysosome are two main regulatory pathways for protein degradation. In HSV-1 infection, TRIM14 recruits USP14 to cleave K48-linked ubiquitination of cGAS at K414 [26]. Previous studies reported that HBx targets SMC5/6 for ubiquitylation by the CRL4 HBX E3 ligase and subsequent degradation by the proteasome [29]. In addition, Previous studies have shown that HBx facilitates autophagy via activating death-associated protein kinase and sensitizes cells to starvation-induced autophagy [30]. Here we speculate that a complex interaction between HBx and the host ubiquitin–proteasome system (UPS) and autophagy may be responsible for the degradation of cGAS, but the underlying mechanisms need to be further explored.

Different research groups have demonstrated that cGAS is a highly effective DNA sensor in many ways. The purified cGAS protein can directly bind to DNA molecules in biochemical tests [3]. Protein crystal structure analysis shows that cGAS binds to negatively charged DNA phosphate skeletons through positive and hydrogen bonding on the protein surface, which does not depend on DNA sequences [31]. Fluorescence co-localization found that cGAS and HBx co-localized intracellularly, and co-IP confirmed that HBx interacts with cGAS but not STING. HBx binds to cGAS, which may interfere with DNA recognition by cGAS. On the one hand, this confirms that HBx is the meritorious statesman of HBV in regulating and escaping cGAS-induced innate immunity. On the other hand, it is reasonable to presume that HBx may block cGAS's DNA recognizing.

In summary, Our research reveals that HBx is an effective molecule of HBV regulating cGAS. It remains unknown whether other pathways or other protein components are involved in HBV regulating cGAS. In addition, we did not investigate the specific sites of GAS binding to HBx and the direct effects caused by their binding, nor did we clarify whether there was any correlation between autophagy and ubiquitination regulation of cGAS. Thus, further studies are still required to elucidate the detailed mechanisms. cGAS debuted as a cytoplasmic DNA sensor. However, recent studies have shown that cGAS is localized mostly in the nucleus [32].

Moreover, cGAS combined with chromatin acts as a decelerator for DNA replication forks, which controls replication dynamics and suppresses replication-associated DNA damage, suggesting that cGAS is an attractive target [33]. cGAS combined with chromatin for exploiting the genomic instability of cancer cells. It remains to be seen whether the chromatin-binding cGAS will interact with the nucleated HBV virus. Further investigation of these questions will certainly shed new light on our understanding of the function of cGAS, which will also help us develop novel therapeutic methods against HBV infection (Table 2).

**Table 2:** Abbreviations

Full name: Abbreviations
cGAS: Cyclic GMP-AMP synthase
HBV: Hepatitis B virus
HBx: Hepatitis B virus X protein
PRRs: Pathogen recognition receptors
IFN-I: Type I interferon
IRF3: Interferon regulatory factor-3
ISGs: IFN stimulated genes
HSV-1: Herpes simplex virus 1
DENV: Dengue virus
KSHV: Kaposi's sarcoma-associated herpesvirus
LANA: Latency-associated nuclear antigen
DMEM: Dulbecco's modified Eagle's medium
pRL-TK: Renilla luciferase
DOC: Deoxycholate
DLR: Dual-luciferase reporter
dsDNA: Double-stranded DNA
K48: Lysine 48
LC3: microtubule-associated protein-Light-chain 3
pcDNA-HBV1.3: HBV 1.3-fold genome plasmid
IFN-β: Interferon beta
TBK1: Tank-binding kinase 1
3-MA: 3-methyladenine
ISG: Interferon-inducible Protein 6–16
ISG56: IFN-stimulated gene 56
ISG54: IFN-stimulated gene 54
WB: Western-Blot

## Conclusion

In conclusion, in this present study, we identified that HBx is an effective molecule of HBV regulating gas. It negatively regulates cGAS-mediated DNA signaling through ubiquitination and autophagy. Studying the role of cGAS in HBV infection provides some new ideas for improving the future treatment of HBV.

## Data Availability

The data and material generated or analyzed in this study are available upon reasonable request and could be provided by Weixian Chen (300801@cqmu.edu.cn).
